# Practical Remarks on Fœtal Auscultation

**Published:** 1860-06

**Authors:** R. Druitt

**Affiliations:** London


*Practical Remarks on Fœtal Auscultation.* By R. Druitt, L. R. C. P., London.—The readers of the *Medical Times* have been startled by the announcement, on the part of one of the most learned physicians in the world, that the fœtal heart cannot be heard before birth; that if certain sounds be heard, said to be those of the fœtal heart, there is no certainty attainable that they are what they are supposed to be; and that the commonly described sounds are illusions, and exist only in the minds of the listeners. First of all, as to the facts asserted, and the reasonableness of them. To many physicians it is absolutely certain, that at most times, in women pregnant of a live child, especially after the fifth month, there can be heard, over some part of the enlarged uterus, a small and distinct, but often remarkably distinct heart-beat, varying from 140 to 180 in the minute. By a heart-beat is meant a double beat, of one louder and more pronounced, and another shorter, immediately succeeding, and less pronounced.

It is said to be incredible that the sounds of the fœtal heart can reach the ear through so great a mass, consisting of uterine tissues, vessels, and the limbs of the child. But the thickness of the uterus and abdominal walls is not great, and not to be compared in sound-deadening qualities to a common pillow. But who doubts that the ticking of a watch can be heard under a pillow? The writer has heard his own watch through the thickness of sixteen folds of blanket. There is, also, no real difficulty concerning the counting of so large a number of pulsations as 160 in a minute. The breathing of dying children may be distinguished at 180; the pulse at 240. The ticks of a lever watch are five to a second, and may most easily be counted at 240 per minute. When the piano is played rapidly, the ear can readily recognize 720 sounds in a minute.

There is no doubt that a woman may be pregnant of a living child, and yet that at times the fœtal heart-sounds not be discovered even by one accustomed to the search. Hence, the absence of the sounds can never be taken as a proof that the fœtus is dead, or that pregnancy does not exist.

Again, sounds may be feebly heard, so that the observer cannot say that they are not heart-sounds; or other sounds may be mistaken for those of the fœtal heart. If the sounds are not confined to a small circle, or are synchronous with those of the maternal heart, they should not be suspected to be fœtal.

Of all the signs which distinguish the enlarged uterus from other tumors, none are more valuable than the following: the uterus, like other hollow viscera, has a regular peristaltic motion, continuous throughout pregnancy, (and after delivery,)

and consisting in periodic contractions, which cause a moderate but decided tension of the organ, and are followed by flaccidity and repose. No other tumor, not tympanitic, can do this; and, during the fits of contraction, the shape, dimensions, and outline of the organ are unmistakable. When about to auscultate, gently shampoo or roll the abdominal parietes over the womb, till it becomes hard and resisting. This is the moment for auscultation. Put the stethescope on the womb, and perpendicular to its surface. Search carefully on the horizontal line on a level with the anterior-superior spine of the ilium; beginning on the left side, then a little above and below; if unsuccessful, go to the right side. Take care that the attitude is easy, and produces no rushing sound in the ears.—*Medical Times and Gazette*, January 21, 1860.

